# Effects of a social network intervention on HIV seroconversion among people who inject drugs in Ukraine: moderation by network gender composition

**DOI:** 10.1186/s12954-023-00899-3

**Published:** 2023-11-08

**Authors:** John Mark Wiginton, Robert Booth, Laramie R. Smith, Sajina Shakya, Cristina Espinosa da Silva, Thomas L. Patterson, Eileen V. Pitpitan

**Affiliations:** 1 Division of Infectious Diseases and Global Public Health, Department of Medicine, University of California San Diego, San Diego, USA; 2 School of Social Work, San Diego State University, 5500 Campanile Dr, San Diego, CA 92182 USA; 3 Department of Psychiatry, University of Colorado School of Medicine, Denver, USA; 4 Herbert Wertheim School of Public Health & Human Longevity Science, University of California San Diego, San Diego, USA; 5 School of Public Health, San Diego State University, San Diego, USA; 6 Departments of Psychiatry, University of California San Diego, San Diego, USA

**Keywords:** Women who inject drugs, Ukraine, Social network intervention, Moderation, People who inject drugs, HIV, Gender

## Abstract

**Background:**

Women who inject drugs in Ukraine are disproportionately burdened by HIV. To help address the needs of this population, a greater understanding of how interventions may uniquely benefit women who inject drugs is needed.

**Methods:**

Data come from a randomized controlled trial of a social network intervention targeting people who inject drugs in Ukraine (N = 1195). Indexes, plus two of their injection network members, received HIV testing and counseling (control arm) or HIV testing and counseling plus a social network intervention (intervention arm), in which indexes were trained to influence network members’ risk behaviors. We used Cox regressions with interaction terms to assess differences in time to HIV seroconversion between arms by network gender composition and gender of the index. For significant interaction terms, we calculated simple effects, generated survival functions using Kaplan–Meier methods, and compared survival curves using log-rank tests.

**Results:**

At 12 months, there were 45 seroconversions among women (40.0 [28.3, 51.7] per 100 person years) and 111 among men (28.4 [23.1, 33.6] per 100 person years) in the control arm; there were 27 seroconversions among women (17.1 [10.7, 23.6] per 100 person years) and 77 among men (18.7 [14.5, 22.9] per 100 person years) in the intervention arm. Network gender composition (but not gender of the index) moderated the intervention effect on HIV incidence (*p* < 0.05). Specifically, the intervention appeared to be even more protective against HIV acquisition as female gender composition increased. In the intervention arm, the HIV seroconversion hazard rate was 44% lower with 1 network female; 61% lower with 2 network females; and 72% lower with 3 network females.

**Conclusions:**

A greater number of women in an injection network, coupled with the provision of risk-reduction strategies, is associated with HIV risk-mitigation, though the mechanisms through which this occurs remain unclear. Findings can support new research and practice directions that prioritize women who inject drugs and more thoughtfully support their health and wellbeing.

## Introduction

Globally, people who inject drugs (PWID) are disproportionately burdened by HIV infection, but further disparities exist within the PWID population [1, 2]. An international systematic review of studies with PWID in North and South America, Europe, and Asia concluded that women who inject drugs have a significantly higher prevalence of HIV relative to men who inject drugs, despite comprising only one fifth of the global PWID population [3]. A similar review identified being female as a risk factor for HIV among PWID in Central Asia and Eastern Europe, including Ukraine [4].

Ukraine, a former Soviet republic in Eastern Europe, has the second-highest HIV incidence rate (39.0–42.5/100,000 in 2019) among the 53 countries comprising the WHO European Region [5]. Unsafe injection drug use (IDU) behaviors initially drove the HIV epidemic in Ukraine, and though heterosexual sex is now the dominant route of transmission, unsafe IDU alone continues to account for roughly one in four new HIV infections per year [6, 7]. HIV prevalence has been consistently high among all PWID in Ukraine but has remained higher among women compared to men, though women who inject drugs make up only one in four of Ukraine’s roughly 350,000 PWID [8]. HIV prevalence estimates among women compared to men who inject drugs in Ukraine have ranged from 14–40% versus 14–32% [3] and 22–31% versus 17–22% [9], with an HIV prevalence rate ratio of 1.25 (95% CI = 1.16, 1.34; 22.6% vs 18.1%) [10]. Moreover, women who inject drugs in Ukraine have been shown to make up a higher proportion of individuals newly diagnosed with HIV across diverse recruitment and testing strategies [11].

Factors across socioecological levels may amplify HIV risk for women who inject drugs in Ukraine. For example, individual-level factors include low condom use (44%) among women and men who inject drugs, and women’s enhanced biological susceptibility to contract HIV sexually [2, 12, 13]. Interpersonal and social network-related factors include syringe/needle-sharing with an injecting male partner, and violence and forced sex work by a male injecting partner [1, 2, 12, 14–17]. Community- and structural-level factors include sexist, patriarchal values and intersecting gender, HIV, and drug/IDU-related stigmas that cut across all other socioecological levels and undermine women’s agency and power further, affecting access to HIV and drug treatment [14, 16, 18–24].

Of note, however, is that protective factors have also been identified, with one social network factor in particular, gender homophily—the extent to which network members are the same gender—emerging as potentially critical to mitigate injection risk in this population. Having more women in one’s network has been shown to facilitate reductions in unsafe injection behaviors [25], increase awareness of HIV pre-exposure prophylaxis [26], lower frequency of arrests [27], and be associated with greater social support [28]. More systematic attention on network gender composition may therefore be pertinent to understanding and improving the health and psychosocial wellness of this specific sub-population.

Individual-level and social network/peer-led interventions have been implemented among PWID in Ukraine, with varying degrees of efficacy [29–32]. However, few interventions in Ukraine have been tailored to women who inject drugs, despite the disproportionate HIV risk faced by this group, and despite evidence that social network interventions may benefit women who inject drugs more than men who inject drugs [33]. Given the precarity of women who inject drugs’ social position and amplified HIV risks in Ukraine [7, 25, 34, 35], re-examining past intervention trial data to determine differences in efficacy by gender is a critical next step toward motivating and informing the development of interventions tailored to women who inject drugs in Ukraine, as well as comparable contexts in the region (such as other former Soviet blocs). Evaluating intervention trial data using nuanced approaches (e.g., considering multiple operational definitions of a potential effect modifier) is imperative to reach a fuller, more detailed understanding of intervention efficacy (e.g., identification of subgroups for whom the intervention was more or less effective). Moreover, maximizing use of available data is responsible, efficient, and cost-effective given how labor- and resource-intensive intervention trial implementation is [36].

A randomized controlled trial evaluated a social network intervention that trained PWID in Ukraine to educate their injection network members about safe injection and sexual practices, and this intervention was ultimately found to reduce HIV incidence [31]. However, this finding was based on the effect being averaged over all participants; other analyses, such as moderation or effect modification by gender, were not explored. The objectives of this secondary analysis were to determine whether the effect of the intervention on HIV seroconversion was moderated by network gender composition and by index gender (i.e., gender of the index). Though the data were collected roughly ten years ago, recent data show that women who inject drugs continue to be disproportionately burdened by HIV infection in Ukraine [9–11]. Also, while the extent to which the Russian invasion may change the epidemiologic context of HIV and IDU in Ukraine is unclear, disruption of HIV prevention efforts and exacerbation of gender disparities in HIV-related health outcomes can be expected [37], underscoring the urgency of understanding how best to intervene among women who inject drugs in Ukraine moving forward.

## Methods

### Data source, participants, and procedures

Data were drawn from a randomized controlled trial of a social network intervention targeting PWID in Odesa, Donetsk, and Mykolaiv, Ukraine. Detailed methods have been described elsewhere [31]. Briefly, the intervention sought to train peer leaders, or index participants, as educators to influence the injection and sexual risk behaviors of their injection network members. Index participants were recruited from the streets by outreach workers from nongovernmental organizations in each of the three cities. Eligibility criteria included being 16 years of age or older, self-reported drug injection in the past 30 days (verified by signs of recent venipuncture), willingness to participate in interviews and HIV testing, ability to provide informed consent, and willingness to recruit two members of their injection network for participation in the study. Network members recruited by index participants also had to meet these eligibility criteria (except for willingness to recruit two members). Recruitment lasted from July 2010 to November 2012.

The intervention arm received Ukraine’s standard of care HIV testing and counseling, an updated version of the Counseling and Education model developed during the National Institute on Drug Abuse’s cooperative agreement, and the social network intervention based on SHIELD (Self-Help in Eliminating Life-Threatening Diseases) [38–40], in which index participants were trained to teach their injection network members how to reduce HIV risk behaviors. Training occurred in five small-group sessions over two weeks and involved teaching peer leaders how to model and discuss safe behaviors via role-playing and other techniques. Network members assigned to the intervention arm received no training or additional intervention, as the social network intervention was based on index participants providing peer education to their injection network members. The control arm received HIV testing and counseling only. The present analysis included HIV negative participants only (N = 1200). The intervention arm consisted of 611 participants, including 190 indexes; the control arm consisted of 589 participants, including 171 indexes. All participants were interviewed and HIV-tested at baseline, 6 months, and 12 months.

### Measures

The primary outcome was time to HIV seroconversion. HIV seroconversion at any time point between baseline and 12 months was considered an incident HIV infection, and date of seroconversion was estimated as the midpoint between the participant’s last HIV negative result and their HIV positive result.

Interviewers were instructed to code the gender of participants as male (coded “0”) or female (coded “1”) at baseline. Admittedly, this was not a true assessment of gender, as it was based on the perception and judgment of the interviewer and conflated, perhaps inadvertently, sex with gender. However, for the purposes of this analysis, we call this variable *gender* and consider those coded male to be men and those coded female to be women.

We examined gender as a potential moderator of the effect of the intervention on HIV seroconversion, which we operationalized and tested in two ways. First, network gender composition (i.e., number of women), including the index (range = 0–3), was assessed to determine if the effect of the intervention differed by the number of women comprising the network. This scenario would permit both female indexes and female network members to be included in the moderation effect. Second, moderation by index gender was assessed to determine if the effect of the intervention differed by whether or not the index was female, regardless of network gender composition. We examined number (rather than proportion) of women due to low variability in network size. Control variables were consistent with those in the original study and included age (continuous), city (Odesa vs Donetsk; Mykolaiv vs Donetsk), and injection frequency at baseline (continuous), though we log-transformed injection frequency to stabilize variance.

### Analysis

We calculated descriptive statistics for sociodemographic characteristics and other variables of interest and used chi-square tests (for categorical covariates) and Kruskal–Wallis tests (for continuous covariates) to assess differences in sociodemographic and network characteristics between intervention and control arms.

We calculated hazard ratios (HR) with 95% confidence intervals (CI) by employing Cox regression analysis (with a frailty term to fit a random intercept for peer networks) to assess the extent to which time to HIV seroconversion between intervention and control arms differed by network gender composition and index gender. Model 1 included an interaction term between study arm (intervention vs control) and network gender composition, including index (female vs male); and Model 2 included an interaction term between study arm (intervention vs control) and index gender (female vs male). For significant interaction terms, we calculated simple effects to explore the nature of the interaction and generated survival functions stratified by experimental arm using Kaplan–Meier methods to plot and compare time to failure (i.e., HIV seroconversion). We compared corresponding survival curves (e.g., intervention participants with 2 network females compared to control participants with 2 network females) using log-rank tests.

We used several methods to assess the proportional hazards assumption. We graphed separate Kaplan–Meier survival functions for each categorical covariate. Visual inspection of the graphs showed parallel survival functions that diverged slowly over time and did not cross. We plotted scaled Schoenfeld residuals by time for all covariates and inspected the cumulative sum of residuals (score process pattern) with the corresponding results for a random selection of 20 out of 1000 simulated score process patterns. We then performed Kolmogorov-type supremum tests for all covariates to assess departure of the observed score process from the simulated ones and found none to be significant. Next, we generated time-dependent covariates by creating interaction terms between survival time and each covariate and added them to the models and found none to be significant. Finally, linear hypothesis testing and the score test yielded non-significant results. All of these procedures indicated that the proportional hazards assumption held. We calculated descriptive statistics in Stata (StataCorp, College Station, TX) and performed all other analyses in SAS (SAS Institute, Cary NC).

## Results

Five network participants were missing data indicative to which network they belonged and were excluded, leaving 1,195 participants in the present analysis. Comparable proportions of participants were recruited in Odesa (n = 421; 35.2%) and Mykolaiv (n = 411; 34.4%), followed by Donetsk (n = 363; 30.4%). Mean age was 31.3 years (SD = 8.4), and three in four participants were men (n = 894). Three in four participants were Ukrainian (n = 900), and more than 20% were Russian (n = 258). No baseline differences in sociodemographic characteristics between conditions were found (Table 1).

**Table 1:** Baseline sociodemographic and network characteristics of PWID in Ukraine (N = 1195)

	Control arm (n = 585)	Intervention arm (n = 610)	Overall/Total (N = 1195)
Age in years			
Mean (SD)	31.4 (8.8)	31.2 (7.9)	31.3 (8.4)
Median (IQR)	30 (25–37)	30 (25–36)	30 (25–37)
*χ*^2^ (*p*-value)	–	–	0.01 (0.925)
Gender, n (%)			
Female	136 (23.2)	165 (27.0)	301 (25.2)
Male	449 (76.8)	445 (73.0)	894 (74.8)
*χ*^2^ (*p*-value)	–	–	2.29 (0.130)
Ethnicity, n (%)			
Ukrainian	427 (73.0)	473 (77.5)	900 (75.3)
Russian	137 (23.4)	121 (19.8)	258 (21.6)
Other	21 (3.6)	16 (2.6)	37 (3.1)
*χ*^2^ (*p*-value)	–	–	3.50 (0.174)
City of residence, n (%)			
Odesa	205 (35.0)	216 (35.4)	421 (35.2)
Mykolaiv	193 (33.0)	218 (35.7)	411 (34.4)
Donetsk	187 (32.0)	176 (28.9)	363 (30.4)
*χ*^2^ (p-value)	–	–	1.62 (0.445)
Relationship status, n (%)			
Single	288 (49.2)	286 (46.9)	574 (48.0)
Married	85 (14.5)	89 (14.6)	174 (14.6)
Common law married, cohabiting	93 (15.9)	123 (20.2)	216 (18.1)
Separated, divorced	95 (16.2)	92 (15.1)	187 (15.6)
Widowed, other	24 (4.1)	20 (3.3)	44 (3.7)
* χ*^2^ (*p*-value)	–	–	4.16 (0.385)
Injection frequency^a^			
Mean (SD)	3.0 (0.9)	3.1 (0.9)	3.0 (0.9)
Median (IQR)	3.0 (2.4–3.7)	3.1 (2.3–3.7)	3.1 (2.4–3.7)
*χ*^2^ (*p*-value)	–	–	0.40 (0.525)
Gender of network members^b^			
Female	107 (25.8)	116 (27.6)	223 (26.7)
Male	307 (74.2)	304 (72.4)	611 (73.3)
*χ*^2^ (*p*-value)	–	–	0.33 (0.566)
Number of women in network, including index			
0	252 (43.1)	268 (43.9)	520 (43.5)
1	240 (41.0)	220 (36.1)	460 (38.5)
2	81 (13.8)	92 (15.1)	173 (14.5)
3	12 (2.1)	30 (4.9)	42 (3.5)
*χ*^2^ (*p*-value)	–	–	9.26 (0.026)*
Number of women in network, excluding index			
0	310 (53.0)	326 (53.4)	636 (53.2)
1	223 (38.1)	223 (36.6)	446 (37.3)
2	52 (8.9)	61 (10.0)	113 (9.5)
*χ*^2^ (*p*-value)	–	–	0.60 (0.742)
Index gender^c^			
Female	29 (17.0)	49 (25.8)	78 (21.6)
Male	142 (83.0)	141 (74.2)	283 (78.4)
*χ*^2^ (*p*-value)	–	–	4.14 (0.042)*

PWID, people who inject drugs; SD, standard deviation; IQR, interquartile range

^a^Log-transformed

^b^Out of a total of 834 network members (control: n = 414; intervention: n = 420)

^c^Out of a total of 361 indexes (control: n = 171; intervention: n = 190)

*p < 0.05

Both the control and intervention arms had networks comprised of comparable proportions of women when excluding indexes (*χ*^2^[2] = 0.60, *p* = 0.742) but not when including indexes (*χ*^2^[3] = 9.26, *p* = 0.026). In the control arm, 1 in 6 indexes were women; in the intervention arm, 1 in 4 indexes were women (*χ*^2^[1] = 4.14, *p* = 0.042). Since our aim was to target gender as a moderator of intervention effects, the models we tested inherently adjusted for this difference between study arms. At 12 months, there were 260 HIV seroconversions over 1,073.4 person years, including 72 women (26.7 [20.5, 32.8] per 100 person years) and 188 men (23.4 [20.1, 26.7] per 100 person years). In the control arm, there were 45 seroconversions among women (40.0 [28.3, 51.7] per 100 person years) and 111 among men (28.4 [23.1, 33.6] per 100 person years). In the intervention arm, there were 27 seroconversions among women (17.1 [10.7, 23.6] per 100 person years) and 77 among men (18.7 [14.5, 22.9] per 100 person years) (Table 2).

**Table 2:** Main and interaction effects of female gender and intervention arm on HIV incidence among PWID in Ukraine (N = 1195)

	HR (95% CI)	aHR^a^ (95% CI)
Model 1		
Network gender composition	1.20 (0.96, 1.51)	1.13 (0.93, 1.38)
Study arm (intervention vs control)	0.75 (0.51, 1.09)	0.79 (0.56, 1.11)
Interaction	0.72 (0.52, 1.02) ~	0.71 (0.52, 0.97)*
Model 2		
Index gender (female vs male)	1.17 (0.56, 1.77)	1.10 (0.61, 1.63)
Study arm (intervention vs control)	0.61 (0.45, 0.82)**	0.62 (0.47, 0.83)**
Interaction	0.88 (0.47, 1.64)	0.85 (0.47, 1.56)

HIV, human immunodeficiency virus; PWID, people who inject drugs; HR, hazard ratio; aHR, adjusted hazard ratio; CI, confidence interval

^a^Controlling for age, city, log-injection frequency, and main effects of study arm and gender variable

~ *p* < 0.10 *p < 0.05, **p < 0.01, ***p < 0.001

### Network gender composition

**Fig. 1:**
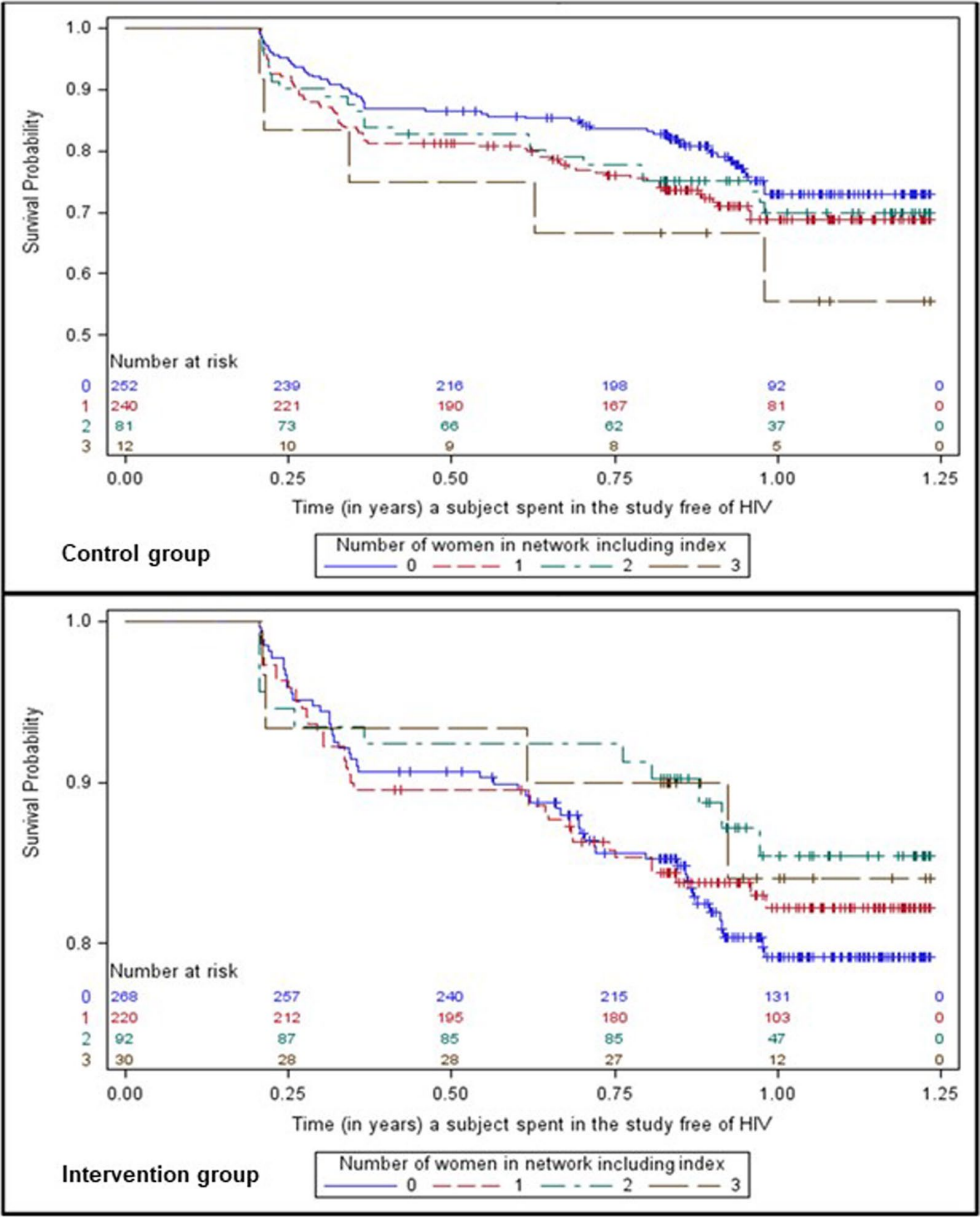
Kaplan–Meier product-limit survival estimates of time to HIV seroconversion by number of females in one’s injection network, including index. HIV, human immunodeficiency virus

The main effect of network gender composition was non-significant in unadjusted and adjusted models (*p* = 0.109 and *p* = 0.222, respectively), as was the main effect of study arm (*p* = 0.135 and *p* = 0.170, respectively) (Table 2). The interaction was significant in the adjusted model (*p* = 0.031). Simple effects indicated that, in the control arm, for each additional network female, the HIV seroconversion hazard rate was 13% higher (HR = 1.13; 95% CI = 0.93, 1.38; *p* = 0.222). In the intervention arm, for each additional network female, the HIV seroconversion hazard rate was 20% lower (HR = 0.80; 95% CI = 0.63, 1.03; *p* = 0.078). In the intervention arm, the HIV seroconversion hazard rate was 21% lower with 0 network females (HR = 0.79, 95% CI = 0.56, 1.11), which was non-significant (*p* = 0.170); 44% lower with 1 network female (HR = 0.56, 95% CI = 0.43, 0.73), which was significant (*p* < 0.001); 61% lower with 2 network females (HR = 0.39, 95% CI = 0.25, 0.63), which was significant (*p* < 0.001); and 72% lower with 3 network females (HR = 0.28, 95% CI = 0.13, 0.59), which was significant (*p* < 0.001). Survival curves for time to HIV seroconversion by number of network females stratified by study arm are presented in Fig. 1. Log-rank tests indicated that time to HIV seroconversion was significantly longer for intervention participants with 1 or 2 network females compared to control participants with 1 or 2 network females (*p* = 0.002 and *p* = 0.0.015, respectively), and marginally longer for intervention participants with 3 network females compared to control participants with 3 network females (*p* = 0.057).

### Index gender

The main effect of index gender was non-significant in both unadjusted and adjusted models (*p* = 0.656 and *p* = 0.451, respectively), while the main effect of study arm was significant in both (both *p* = 0.001). The interaction term between study arm and index gender was non-significant in the adjusted model (*p* = 0.605). Consequently, we did not calculate simple effects or generate survival curves.

### Sensitivity analysis 1: testing network gender composition excluding the index

Given the significant interaction effect when network gender composition including the index was examined, we sought to conduct a sensitivity analysis to examine whether this pattern of results would hold if we excluded the index. We found that the main effect of network gender composition excluding the index was non-significant in unadjusted and adjusted models (*p* = 0.147 and *p* = 326, respectively), as was the main effect of study arm (*p* = 0.082 and *p* = 0.096, respectively). However, their interaction was significant in both the unadjusted (*p* = 0.032) and adjusted models (*p* = 0.034), lending further support for the role of network gender composition. Simple effects indicated that, in the control arm, for each additional network female, the HIV seroconversion hazard rate was 13% higher (HR = 1.13; 95% CI = 0.88, 1.45), but this was non-significant (*p* = 0.326). In the intervention arm, for each additional network female, the HIV seroconversion hazard rate was 26% lower (HR = 0.74; 95% CI = 0.54, 1.01), which approached statistical significance (*p* = 0.059). In the intervention arm, the HIV seroconversion hazard rate was 24% lower with 0 network females (HR = 0.76, 95% CI = 0.55, 1.05), which was non-significant (*p* = 0.096); 51% lower with 1 network female (HR = 0.49, 95% CI = 0.36, 0.68), which was significant (*p* < 0.001); and 68% lower with 2 network females (HR = 0.32, 95% CI = 0.17, 0.61), which was significant (*p* < 0.001).

### Sensitivity analysis 2: moderation by network gender, stratified by gender

To further examine our findings regarding network gender composition, we conducted a sensitivity analysis to test whether male and female network members varied in response to the intervention by the number of females present in the injection network. We repeated our Model 1 analysis, in which we incorporated a product term between study arm and network gender composition in our Cox regression, but we also stratified by gender. We calculated simple effects regardless of the significance of the product terms to examine trends.

**Fig. 2:**
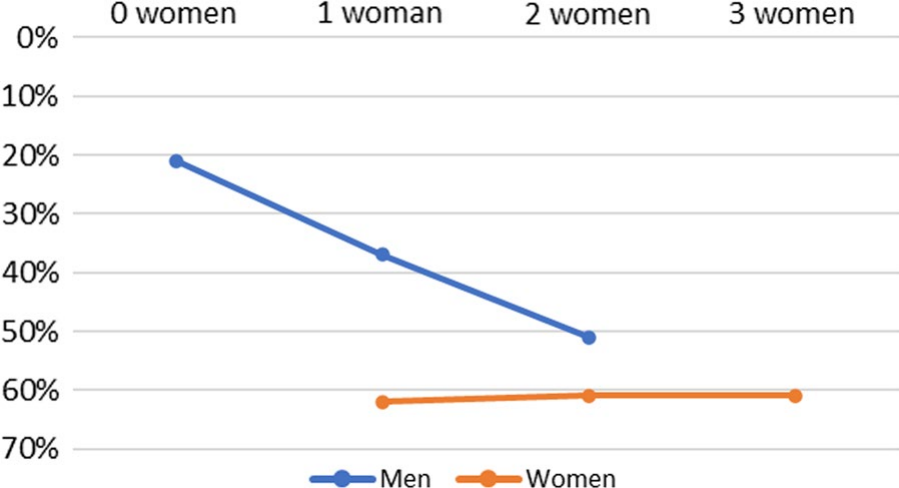
Reductions in HIV seroconversion risk by number of women in one’s injection network for intervention vs control participants, stratified by gender. HIV, human immunodeficiency virus

For men (n = 894) and women (n = 301), product terms were non-significant in both unadjusted (*p* = 0.195 and *p* = 0.820) and adjusted (*p* = 0.330 and *p* = 0.981) models, respectively. Among men in the intervention arm, the HIV seroconversion hazard rate was 21% lower with 0 network females (HR = 0.79, 95% CI = 0.55, 1.14), which was non-significant (*p* = 0.213); 37% lower with 1 network female (HR = 0.63, 95% CI = 0.42, 0.92), which was significant (*p* = 0.018); and 51% lower with 2 network females (HR = 0.49, 95% CI = 0.22, 1.09), which was marginally significant (*p* = 0.079). Among women in the intervention arm, the HIV seroconversion hazard rate was 62% lower with 1 network female (HR = 0.38, 95% CI = 0.19, 0.78), which was significant (*p* = 0.009); 61% lower with 2 network females (HR = 0.39, 95% CI = 0.19, 0.77), which was significant (*p* = 0.007); and 61% lower with 3 network females (HR = 0.39, 95% CI = 0.10, 1.52), which was non-significant (*p* = 0.173; Fig. 2).

## Discussion

In this study, we explored the extent to which network gender composition and index gender moderated the effects of a social network intervention on HIV seroconversion among PWID in Ukraine. Though women comprised only one quarter of the sample, our findings revealed significant moderation by network gender composition but not by index gender. Our first sensitivity analysis also revealed significant moderation by network gender composition even though we excluded the index, while our second sensitivity analysis showed a sustained, constant trend of reduced risk among women and a decreasing trend of reduced risk among men as the number network females increased. These findings contribute to the broader literature that has shown social network approaches to be effective in documenting and reducing unsafe behaviors among PWID across diverse contexts, including Ukraine [30, 38, 41–50]. More importantly, these findings extend prior research, particularly research in Eastern Europe, by contributing to the knowledge base on social network interventions and on *women* who inject drugs specifically, which can help inform the tailoring of future interventions to this group and motivate research to examine mechanisms (e.g., gendered power dynamics) linking gender composition in injection networks to HIV risk [25, 27, 28].

Our main analysis (and first sensitivity analysis) revealed a more robust intervention effect on HIV seroconversion among participants with more women in their injection network, which recalls prior research showing a protective effect for PWID with a greater proportion of women in their networks [25, 27, 28]. One potential explanation for this finding is that participants in these networks were less exposed to gendered (i.e., unequal) power relations and consequently had increased agency to adopt the intervention on account of their being fewer men in the network [25]. Similar to those of women who inject drugs in other contexts [1, 7, 17, 51–57], the injection behaviors and sexual relationships of women who inject drugs in Ukraine are complexly interconnected, with male injecting partners often wielding power over women that increases their HIV risk through unsafe practices (such as syringe/needle-sharing or condomless sex) or limited access to drug treatment (such as opioid agonist treatment) [14, 15]. Arguably, the control participants in networks with comparable gender composition would have likewise experienced reduced exposure to gendered power relations and therefore increased agency. However, they were not presented with safer, alternative risk-reduction practices upon which to enact their agency as were the participants in the intervention arm.

Other explanations for the moderation effect could be related to features of women’s injection networks. Research in other contexts has indicated that the injection networks of women who inject drugs tend to be relatively small and built on trust and close bonds with others [58–61], which may also be characteristic of women who inject drugs in Ukraine [29, 30, 62]. Moreover, women—particularly in traditional, patriarchal societies like Ukraine—tend to be socialized toward engaging in more prosocial behavior than men [63, 64], a tendency also evident in injection networks [59, 60, 65]. Taken together, women in injection networks with more women may be more likely to give (i.e., on the part of the prosocial index member providing education) and receive help (i.e., on the part of network members who are closely bonded with and trust the index), translating to greater uptake of risk-reduction strategies. This is an area for future research.

Our second sensitivity analysis showed support for what we found in our main analysis, demonstrating that men benefited more from the intervention as the number of women in their injection network increased. While women were shown to have benefited regardless of whether other women were in their network, it should be recalled that our sample size of women was a third of our sample size of men, and therefore more prone to parameter estimation error. Nevertheless, findings from both the main and sensitivity analyses do suggest that women uniquely benefited from the intervention and may have even played a role in helping facilitate men’s benefit as well. Again, whether this was due to women’s unique contribution to the network or women’s self-selecting into networks that are inherently safer is unclear, but this lack of clarity on the mechanism does not negate the fact that the presence of women in the network seems to signal greater potential for safer behaviors and reduced HIV risk. Future research should closely examine the mechanisms explaining the role of women in injection risk networks (i.e., whether women promote safer behavior, and/or they self-select into safer networks).

Importantly, we did not assess the full extent of the size or gender composition of participants’ real-world injection networks, as the very nature of the intervention dictated that participants only recruit two of their injection network members. Therefore, participants’ actual injection networks could have been much larger and differently composed than what was indicated in our findings. Unmeasured network links could produce a confounding effect, especially if women’s injection networks vary substantially from those of men [66]. Additionally, the number of women in a given injection network will at least depend on network size, which is associated with HIV risk [67, 68]. Replication trials that fully assess network size, gender composition, and gender dynamics are needed.

There was no moderation effect by index gender. Given women’s more subjugated social position relative to men in Ukraine [14, 21–23, 69–71], including in injection dyads/networks [14, 16], it may be reasonable to expect that having a female index (i.e., be taught risk-reduction strategies by a woman) would be less protective than having a male index. However, our findings did not reveal this to be the case. Given the small number of female indexes in our sample, more targeted research focused on recruiting networks with women indexes are needed to ensure sufficient power to better understand the role of index gender in social network interventions to reduce HIV risk among PWID.

### Limitations

Findings should be considered in light of several limitations. Participants were not asked to report their sex assigned at birth or their gender identity. Rather, interviewers recorded a participant as “male” or “female” based on appearance. Therefore, some participants’ gender identity could have been misreported. Second, as noted previously, conclusions about the overall size or gender composition of participants’ injection networks beyond what was represented in the study cannot be drawn, as participants recruited only two (i.e., not all) members of their injection networks to join the study, and information about their broader networks was not collected. Indeed, had participants’ full injection networks been included, then our findings may certainly differ. Researchers may wish to consider the feasibility of including a sample’s full injection network in future intervention studies. Though the proportion of women who participated in the study was comparable to nationwide population estimates of PWID [35], the unique challenges and disproportionate HIV burden faced by women warrant oversampling of this population to ensure their needs are better understood and addressed.

### Research and practice implications

As this is now one of several studies demonstrating protective effects of having more women in injection networks [25, 27, 28], investigations of the mechanisms (e.g., unequal power relations and other gender dynamics; collective/within-group social support) through which this occurs are needed. Similarly, given that our findings indicated a dose–response relationship of sorts (i.e., the protective effect increased with each additional network female), understanding what the critical share or proportion of network females is needed for maximum, sustained impact on the injection network would be helpful. Because HIV incidence was high in the intervention group as well, despite the intervention’s demonstrated efficacy, full uptake of risk-reduction strategies was not achieved. Teasing out the extent to which there were gender differences in successfully teaching (on the part of indexes) and taking-up risk-reduction strategies (on the part of the network members) would help refine and inform future interventions for women who inject drugs.

Considerations for implementation of this or comparable social network interventions may include a preliminary assessment of gender composition of the injection networks in targeted communities. Informed by these data, organizations may then roll out the intervention to networks with a critical share of network females for optimal intervention impact (though research has yet to determine what this critical share might be, as noted previously). Of course, this is under the assumption that recruited networks in our study, including their size and gender composition, roughly approximate actual networks. Secondly, while a logical implication of our findings would be to alter the gender composition of injection networks, this is likely not an appropriate or feasible course of action. Instead, different sorts of interventions, such as gender-transformative interventions [72, 73], may actually be needed for networks in which the proportion of women is minimal, or for networks with strong gendered or sexist power dynamics. Perhaps in conjunction with, or subsequent to, implementation of gender-transformative programming, our social network or comparable interventions may be implemented. Finally, our finding that the intervention seemed to uniquely benefit women reflects the unique lived experiences of women, including their drug-using experiences, warranting the development of more interventions that are women-focused and women-tailored [74–77]. Of course, future implementation science research would need to be conducted to examine the feasibility, acceptability, and sustainability of the aforementioned intervention strategies.

Implications for further intervention implementation in Ukraine are less clear. As noted previously, these data were collected roughly ten years ago and the applicability to the current Ukrainian context is unknown. Some research conducted during the intervening period suggests that the epidemiologic context of HIV and injection drug use did remain comparable over time [9–11]. However, there is evidence that major recent and current events like the COVID-19 pandemic and the Russian invasion have disrupted HIV prevention and care, drug treatment, and overall healthcare, and will continue to do so [78–82]. These disruptions will likely exacerbate gender disparities in HIV and injection drug-related health outcomes further, as we have seen in previous instances of Russian aggression toward Ukraine [37]. It is perhaps more likely that extant HIV-related disparities faced by women who inject drugs will only grow rather than shrink in response to these events. Therefore, social network interventions like the one examined here, or intentionally women-focused interventions, will continue to remain relevant and needed for women who inject drugs in Ukraine. While the practicalities of successfully implementing any public health intervention in Ukraine in the midst of warfare are formidable, taking the lessons learned from existing data will be valuable as the research and service infrastructure in the country regrow.

## Data Availability

The data that support the findings of this study are available from the authors upon reasonable request and with the permission of the PI.
